# The impact of African swine fever news sentiment on the Korean meat market

**DOI:** 10.1371/journal.pone.0286520

**Published:** 2023-06-30

**Authors:** Byung Min Soon, Wonseong Kim

**Affiliations:** 1 Department of Agricultural Economics, Chungnam National University, Daejeon, Korea; 2 Department of Economics and Statistics, Korea University, Seoul, Korea; Bucharest University of Economic Studies: Academia de Studii Economice din Bucuresti, ROMANIA

## Abstract

Our study analyzed the impact of African swine fever (ASF) news on the Korean meat market using sentiment analysis. We applied a neural network language model (NNLM) to generate a sentiment index indicating whether the news had a positive or negative impact on consumer expectations. We analyzed 24,143 news articles to estimate the impulse responses of meat price variables to sentiment shocks. Our study contributes significantly to agricultural economics as it applies NNLM to generate a sentiment index. The empirical results indicated that ASF news sentiment has a substantial impact on meat prices in Korea, and there is evidence of substitution effects among different types of meat. ASF news has a positive impact on the price of pork, negative effects on beef and chicken prices, and a greater impact on the price of chicken than beef. The findings imply that the effect of ASF news on demand outweighs its impact on supply in the pork market, whereas the effect on supply surpasses the effect on demand in the beef and chicken market. We believe our methods and results will inspire discussions among applied economists studying consumer behavior in this specific market and could encourage the application of big data analysis to the agricultural economy.

## Introduction

News of animal diseases significantly shapes consumer behavior. Consumers can easily obtain information about animal disease outbreaks such as bovine spongiform encephalopathy (BSE) and African swine fever (ASF), through news and social media. They then decide whether to continue consuming meat, or substitute one kind of meat with another. According to economic theory, news may have an impact on meat prices, in addition to the price of meat itself, the price of its substitutes, and consumer income. The volume and intensity of news sentiments change consumers’ expectations of the market situation [1]. Therefore, determining the extent to which news information influences the market is important when studying the impact of animal diseases.

**Fig 1:**
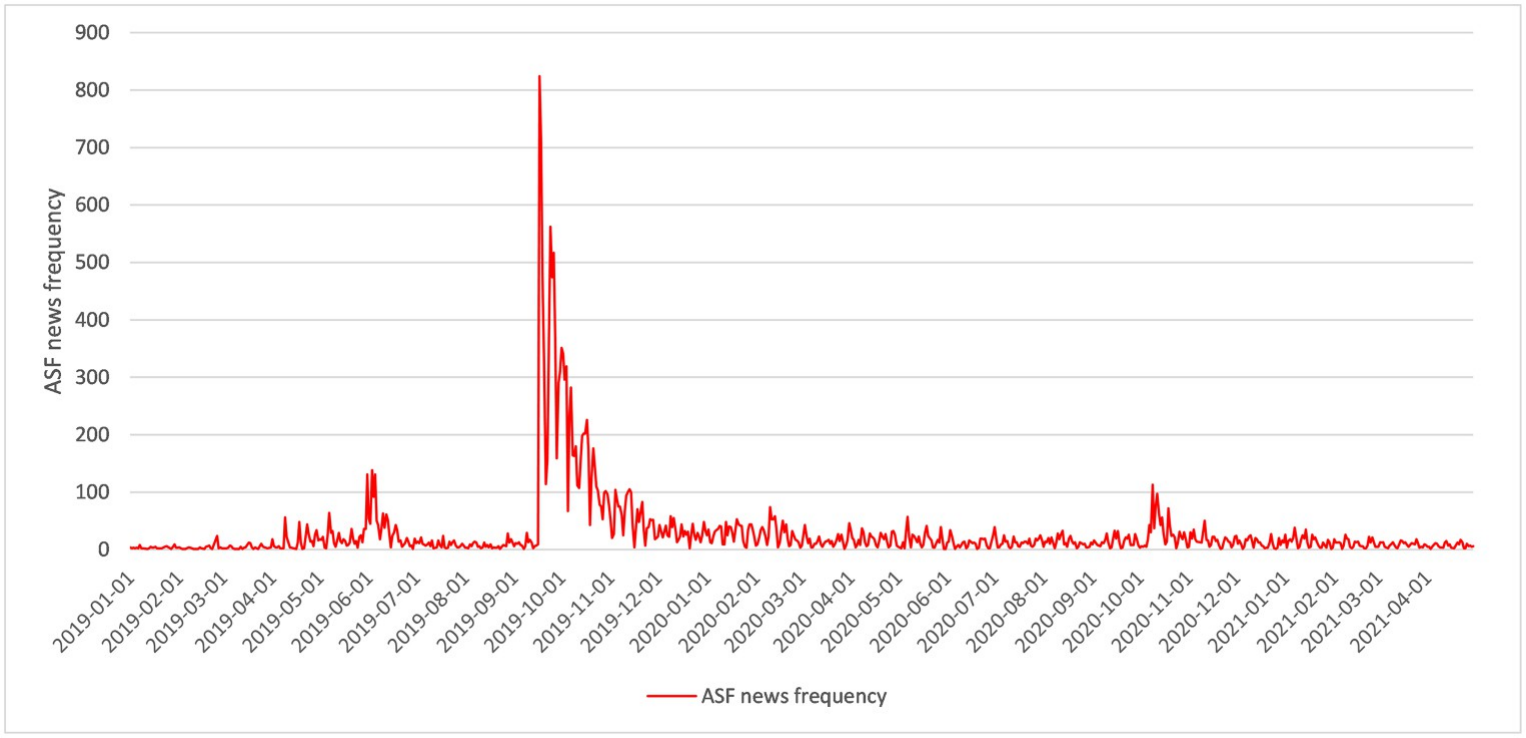
The ASF news frequency in Korea. Source: BIGkinds provided by the Korea Press Foundation (www.bigkinds.or.kr).

Since the first Korean ASF outbreak in September 2019, the volume of news on ASF has drastically increased. Fig 1 shows the ASF news frequency, where we calculated the number of news articles related to ASF. Consumers’ health-related concerns might have changed their behavior regarding consuming pork [2, 3], and they are more likely to consume beef and chicken, rather than pork. The change in the pattern of meat consumption causes an increase in meat price. One possible outcome of the ASF outbreak is a decrease in pork prices due to reduced consumer demand for pork, possibly stemming from health-related concerns. On the other hand, consumers could have substituted pork with beef or chicken. Hence, there could be an increase in the prices of beef and chicken. On the supply side, because the ASF outbreak caused a change in pork production, we should consider the impact of ASF. However, the news contains both positive and negative information, and it is difficult to characterize ASF news as either good or bad.

Big data analysis scraps specific news data and generates a useful index for investigating markets. Machine learning (ML) methods such as word2vec and bidirectional encoder representations from transformers (BERT) have different abilities. Using these ML methods, Loia and Senatore [4] performed traditional sentiment analysis to capture the positive or negative valence of a mood. Wu et al. [5] calculated the real-valued sentiment index of texts using a dimensional sentiment analysis (DSA). In our study, sentiment analysis allowed us to capture the sentiment of ASF news, whether positive or negative, and its seriousness.

Our study aimed to estimate the impact of ASF-related news information on Korean meat prices. We introduced the theoretical framework and performed two procedures. First, we generated a sentiment index by applying a neural network language model (NNLM). Second, we estimated the impulse responses of meat prices to ASF sentiment shocks. This study makes an essential contribution to the field of sentiment analysis in agricultural economics because it fills in a gap in the literature by exploring the causal effect of sentiment on price and consumption behavior. Therefore, we focus on the involvement of sentiment in economic analysis using a theoretical framework.

The remainder of this paper is structured as follows: The next section introduces the empirical literature on the impact of animal diseases on the agricultural market and sentiment analysis. The third section introduces the theoretical framework and explains the sentiment analysis and impulse response function methods. The data are introduced in the fourth section. The fifth section presents the results, and the final section concludes the paper.

## Empirical literature

Several studies have focused on the impact of news on meat markets. Soon and Thompson [6] estimated the impact of the BSE on the Japanese beef market using a time-varying Armington model. They analyzed a counterfactual situation in which a BSE outbreak had not occurred. Ishida et al. [2] showed that BSE and bird flu outbreaks reduced beef and chicken consumption in Japan, because these outbreaks affected consumer behavior. Kawashima and Sari [3] showed that a BSE outbreak in an exporting country changed the country-of-origin bias and elasticity of substitution in importing countries. These studies have mainly used price as a key factor in estimating the impact of animal disease outbreaks. Recently, several studies have been conducted on the impact of COVID-19 on meat markets. Ramsey et al. [7] examined whether COVID-19 caused disruptions in the U.S. meat market using a time-series analysis. The results found that meat markets were integrated, and unexpected movements were found during the COVID-19 period. Lusk et al. [8] estimated the impact of COVID-19 on beef and pork markets through marketing margins and price spreads. The results showed that COVID-19 shock sensitively affects both price spreads and marketing margins.

News plays an essential role in analyzing consumer behavior. Sentiment analysis is a tool commonly used to identify positive and negative emotions. Using natural language processing (NLP), Shapiro et al. [9] investigated consumers’ positive and negative emotions toward situations, such as financial crises and animal disease outbreaks and conducted sentiment analysis using the federal open market committee meeting transcripts as text. Garcia [10] generated financial market sentiments using information from the financial columns of the New York Times. Baker et al. [11] used newspaper text to measure economic policy uncertainty. These studies focused on the financial market to estimate sentiments in the current economic situation. There is a lack of sentiment analyses related to animal diseases in agricultural markets.

There are several options for measuring sentiments. The traditional approach involves the generation of indices through surveys. The survey method has disadvantages in terms of cost, scope, and timeliness [12]. Recently, NLP text sentiment analysis has been used indirectly. There are ways to rely on predefined lists of works, such as lexical methodologies. Words are assigned a negative or positive score, which is used to calculate sentence sentiment [9, 13]. The second approach is an ML technique, which generates sentiment indices. The ML approach to sentiment analysis automatically learns the sentiment weights of words and combines them to measure a sentiment index [9]. The indirect approach costs less than survey-based sentiment measures.

News-based sentiment measurements can be conducted through economic analysis. Shapiro et al. [9] applied an impulse response function to measure the economic sentiment index. They showed that positive news sentiment causes an increase in consumption and output, but a decrease in price. Barsky and Sims [14] used the University of Michigan’s consumer sentiment index to estimate the impact of a positive sentiment shock on consumption, output, and prices, using the impulse response function at a quarterly frequency.

Our study contributes to the literature using NLP and text sentiment analyses to examine the impact of ASF in Korea. We measured ASF news sentiment and generated a news sentiment index. The impulse response function is used to estimate the impact of ASF news sentiment on the Korean meat market. To the best of our knowledge, this is the first study to implement an ASF news sentiment index for time-series applications.

## Method

### Theoretical framework

ASF news can affect meat prices through the supply and demand responses. This study adopted a formula similar to that proposed by Yu [15]. Price elasticity with respect to ASF news can provide insights into how pork, beef, and chicken prices respond to ASF news.

We presumed that ASF news directly affects pork prices, and indirectly influences beef and chicken prices. Hence, we separated the markets into pork and other markets, with beef and chicken being the latter. For the pork market, we assume that both the demand (D_pt_) and supply (S_pt_) of pork are determined by ASF news (I_t_) and pork price (P_pt_) in formula (1):

$$D_{pt}=D\left(I_{t},P_{pt}\right)$$

and

$$S_{pt}=S\left(I_{t},P_{pt}\right).$$ (1)

Taking a total derivative by formula (2):

$$dD_{pt}=\frac{\partial D_{pt}}{\partial I_{t}}dI_{t}+\frac{\partial D_{pt}}{\partial P_{pt}}dP_{pt}$$

and

$$dS_{pt}=\frac{\partial S_{pt}}{\partial I_{t}}dI_{t}+\frac{\partial S_{it}}{\partial P_{pt}}dP_{pt}.$$ (2)

To generate the equilibrium price, the market equilibrium condition (*dD_pt_* = *dS_pt_*,) is

$$\frac{\partial D_{pt}}{\partial I_{t}}dI_{t}+\frac{\partial D_{pt}}{\partial P_{pt}}dP_{pt}=\frac{\partial S_{pt}}{\partial I_{t}}dI_{t}+\frac{\partial S_{pt}}{\partial P_{pt}}dP_{pt}.$$ (3)

Using formula (3), we obtain the pork price elasticity $\varepsilon_{p_{p},I}$ with respect to ASF news as follows:

$$\frac{dP_{pt}}{{dI}_{t}}=\frac{\frac{\partial D_{pt}}{\partial I_{t}}-\frac{\partial S_{pt}}{\partial I_{it}}}{\frac{\partial S_{pt}}{\partial P_{pt}}-\frac{\partial D_{pt}}{\partial P_{pt}}}$$

and

$$\varepsilon_{p_{p},I}=\frac{dP_{pt}}{{dI}_{t}}\frac{I_{t}}{P_{pt}}=\frac{\frac{\partial D_{pt}}{\partial I_{t}}\frac{I_{t}}{D_{pt}}-\frac{\partial S_{pt}}{\partial I_{t}}\frac{I_{t}}{S_{pt}}}{\frac{\partial S_{pt}}{\partial P_{pt}}\frac{P_{pt}}{S_{pt}}-\frac{\partial D_{pt}}{\partial P_{pt}}\frac{P_{pt}}{D_{pt}}}=\frac{\varepsilon_{D,I}-\varepsilon_{S,I}}{\varepsilon_{S,p_{p}}-\varepsilon_{D,p_{p}}},$$ (4)

where *ε*_*D*,*I*_ and *ε*_*S*,*I*_ are the demand and supply elasticities with respect to ASF news, and $\varepsilon_{S,p_{p}}$ and $\varepsilon_{D,p_{p}}$ are the price elasticities of demand and supply for pork. The denominator of formula (4) is always positive because based on the economic theory, $\varepsilon_{S,p_{p}}$ is positive and $\varepsilon_{D,p_{p}}$ is negative. Therefore, the response of pork prices to ASF news depends on *ε*_*D*,*I*_ and *ε*_*S*,*I*_. If *ε*_*D*,*I*_ ˃ *ε*_*S*,*I*_, the pork price increases in response to positive ASF news. However, if *ε*_*D*,*I*_ ˂ *ε*_*S*,*I*_, the pork price decreases in response to positive ASF news.

The beef and chicken market formulas have a substitution effect. The demand and supply for the beef and chicken markets (i) are determined by the ASF news (I_t_), own price (P_it_), and pork price (P_pt_) as the cross price in formula (5).

$$D_{it}=D\left(I_{t},P_{it},P_{pt}\right)$$

and

$$S_{it}=S\left(I_{t},P_{it},P_{pt}\right).$$ (5)

We followed a total derivative and took the market equilibrium condition as in the pork market calculations. We then derived the price elasticity with respect to ASF news for meat, I, using formula (6).

$$\varepsilon_{p,I}^{i}=\frac{\varepsilon_{D,I}^{i}-\varepsilon_{S,I}^{i}+\left(\varepsilon_{D,p_{p}}^{i}-\varepsilon_{S,p_{p}}^{i}\right)\varepsilon_{p_{p},I}^{i}}{\varepsilon_{S,p}^{i}-\varepsilon_{D,p}^{i}}$$ (6)

where $\varepsilon_{D,I}^{i}$ and $\varepsilon_{S,I}^{i}$ are the demand and supply elasticities, with respect to the ASF news for beef and chicken. $\varepsilon_{S,p_{p}}^{i}$ and $\varepsilon_{D,p_{p}}^{i}$ are the cross-price elasticities of demand and supply for beef and chicken, respectively. According to the economic theory, the denominator of formula (6) is always positive. Additionally, $\varepsilon_{D,p_{p}}^{i}-\varepsilon_{S,p_{p}}^{i}$ in the numerator is also positive because of the cross-effect of demand and supply. Therefore, the response of beef and chicken to ASF news is determined by $\varepsilon_{D,I}^{i},\varepsilon_{S,I}^{i}$, and $\varepsilon_{p_{p},I}^{i}$.

Our theoretical framework introduces the response of meat prices to ASF news. However, we require an ASF variable and an empirical analysis approach using price variables for the response analysis. Therefore, we introduce sentiment analysis to explain how we generate the sentiment score for ASF and time series analysis using the ASF sentiment score and meat prices. Other variables affect prices, such as income and input prices. However, to justify how ASF news affected meat prices, we did not consider other variables as we introduced formula (6).

### Sentiment analysis

The ASF sentiment index extracts and quantifies sentiments intuitively and interprets the model outputs with the researcher’s verification of the entire process. Moreover, unsupervised learning methods have a black-box problem: the model is assessed based on final outputs without considering how the results are obtained [16].

Word2Vec, a well-known NNLM, is used to embed words [17]. It assumes that words with similar distributions have similar meanings. Words that appear together in similar contexts in a sentence can be inferred to have similar meanings and relationships. On the word2vec NNLM we applied “Skip-gram,” a learning method used to predict what context words will be allocated near a target word [17]. Suppose that you have a sliding window of a fixed size moving along a sentence, the word in the middle is the target, and those on its left and right within the sliding window are context words.

Specifically, word2vec embeds words in a vector space and calculates the cosine similarity between the two words using formula (7).

$$similarity=cos\left(\theta \right)=\frac{A∙B}{\left\|A\|\|B\right\|}=\frac{\sum_{i=1}^{n}A_{i}\times B_{i}}{\sqrt{\sum_{i=1}^{n}{{(A}_{i})}^{2}}\times \sqrt{\sum_{i=1}^{n}{{(B}_{i})}^{2}}}$$ (7)

where it measures the similarity between two nonzero vectors of the inner product space and *A*_*i*_ and *B*_*i*_ are the components of vectors word A and B, respectively. The similarity has a value between -1 and 1, with 0 indicating perfect independence. If the value is close to 1, both words are highly relevant to the sentence [18]. The S1 File shows the relevance of the quadrant space.

In recent years, many methods have been developed to deal with NLP problems, such as the Euclidean, cosine, Jaccard, Dice, and Jensen-Shannon divergences. Cosine, which measures the angle between two vectors, is the most popular method [19]. Because this study placed tokenized words in 100-dimension vectors, it was necessary to judge the similarity in a multidimensional space, for which cosine similarity is advantageous [20]. Cosine similarity is a well-known method for determining the directions of the vectors. One of the distance measurements, the Minkowski family, which includes the Euclidean and Manhattan distances, performs well with an isolated or compacted dataset [21]. However, word vectors were treated in 100 dimensions to analyze the entire word in 24,143 articles. Cosine similarity remedies these shortcomings and precisely calculates the similarity between two words.

The cosine similarity captures the orientation of words, rather than their magnitude [22]. In this study, we use the concept of cosine similarity to set the ASF dictionary. This study assumes that referring to negative words can negatively affect consumers regardless of whether the news presents a positive or negative mood (the concept of biased words). Consequently, the negative dictionary presented a high cosine similarity with the ASF. Cosine similarity can reasonably quantify the negative impact of the news. An example of simple embedding performance is provided in the S2 File.

**Fig 2:**
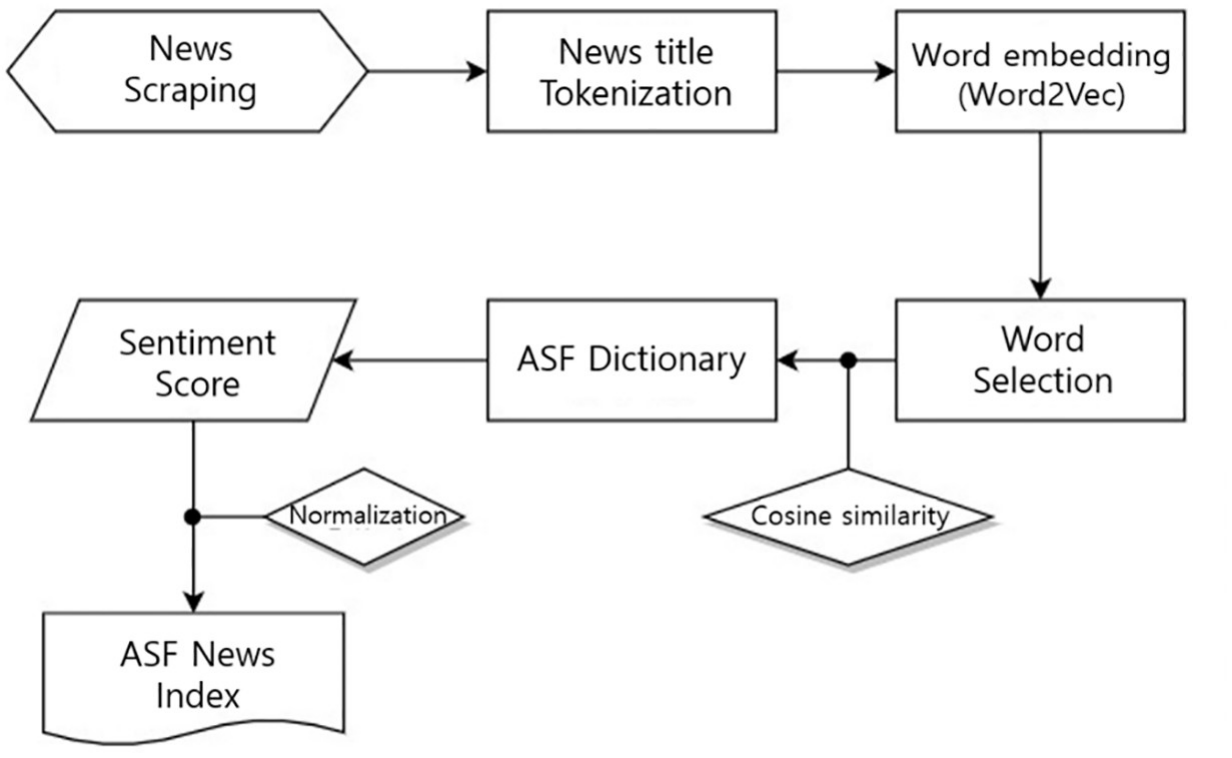
Flow chart for sentiment index.

A six-step procedure was conducted to obtain the sentiment index (Fig 2). Several steps are involved in obtaining the ASF sentiment index.

1. Scrapping 54 news articles from the media platform BIGkinds.
2. Tokenizing news titles with Open Korea Text (OKT), steps two to five are performed using Google Colab.
3. Embedding words using word2vec (vector size = 100, window = 5, min_count = 10, workers = 10, iteration = 200, method = skip-gram).
4. Creating a new lexicon for ASF sentiments. We calculate cosine similarity against “Biased word (ASF).” Words with more than 90% similarity with biased words to be added to new lexicon.
5. The constructed lexicon is called an ASF sentiment dictionary. The ASF lexicon is composed of an existing lexicon (KNU Bi-LSTM, shown in more detail in the [S3 and S4 Files]) and the newly created lexicon. The ASF lexicon is driven by the specific domain, “Animal disease.” Our approach is possibly biased to negative valence, since the ASF lexicon adds new negative words that are similar to “African Swine Fever.” Sometimes, negative words can convey negative perceptions, even if they are used in positive news.
6. Based on the ASF lexicon, this study attempts to determine sentence-level sentiment scores (SC) from word vectors. The overall ASF SC for a news title is defined by the formula (8).
$$SC\left({news\;title}_{i}\right)=\sum_{n=1}^{n}Positve\left(w_{i}\right)-\sum_{k=1}^{k}Negative(w_{i})$$ (8)
  *SC(news title) = sentiment score of *i*^*th*^ news title
  *n* = number of positive words in the *i*^*th*^ news title
  *k* = number of negative words in the *i*^*th*^ news title
  The SC consists of the scores for the words contained in each news title. The maximum length of news titles was 24 tokenized words, which were then sorted by the ASF lexicon as positive or negative. The sorted words mark a score of +1 or -1 each. The sum of the words was the sentence-level sentiment score.
7. The score shows the maximum +5 and the minimum -7. The ASF sentiment is skewed toward a negative valence. Nevertheless, we could not confirm whether the ASF sentiment was more negative than positive. We added the ASF lexicon to precisely determine the depth of the negative impact of disease news. To obtain the results of the ASF sentiment score intuitively, it was normalized between 0 and 1 using the minimum-max method. The closer it is to 1, the more positive is the impact on the market, and the closer it is to 0, the more negative impact to the market.

### Time-series analysis

The impulse response function (IRF), motivated by Shapiro et al. [9], is used to estimate the response of meat prices to ASF news shocks. The IRF in the vector autoregression (VAR) shows how the output variables (beef, pork, and chicken prices) respond to the ASF news shock over time. We built a VAR system incorporating meat prices and the ASF news sentiment index using formula (9):

$$M_{t}=\left[\begin{array}{c} SI_{t}\\ \begin{array}{c} P_{t}\\ \begin{array}{c} B_{t}\\ C_{t} \end{array} \end{array} \end{array}\right]=B_{0}+\sum_{i=1}^{k}B_{i}M_{t-i}+\varepsilon_{t}$$ (9)

where M_t_ is a vector consisting of ASF news sentiment index (SI_t_) and the prices of pork (P_t_), beef (B_t_), and chicken (C_t_). B_i_ is a matrix of coefficients and B_0_ is an identity matrix. The VAR system allows for the estimation of meat price responses to changes in the ASF news sentiment index. The optimal lags are chosen based on the Akaike information criterion (AIC).

The IRF responds to changes in the ASF news on pork, beef, and chicken prices. If consumers and producers perceive positive or negative news about ASF, agencies react to ASF news. If the news is positive, pork consumers are more likely to consume pork than other types of meat. Additionally, producers are likely to produce more pork in response to an increase in expected pork consumption. Moreover, the beef and pork markets function in contrast to positive ASF news. The price could rise or fall, depending on how news changes the demand and supply sides. We can interpret the effect of ASF news sentiments on meat prices, from the IRF results, depending on the aggregate effects of supply and demand in response to ASF news sentiment.

## Data

### News articles for ASF sentiment

The original source of data was the Korean news big data platform BIGkinds, operated by the Korea Press Foundation (www.bigkinds.or.kr). The platform also provided a news scrapping service, that specifies keywords, media types, and geometric classifications. We focused on online daily news in 54 media, between January 2019 and April 2021, which was tagged with either “African Swine Fever (아프리카 돼지열병)” or “ASF.” Summary statistics of the frequencies are shown in [S1 Table]. Most ASF news articles were published between September 2019 and November 2019. At the maximum, on September 17, 2019, people were exposed to 824 online news items for only one day. On average, news media publishes approximately 30 news articles per day.

### Time-series data

Pork, beef, and chicken constitute a major part of meat consumption in Korea. According to the Ministry of Agriculture, Food, and Rural Affairs (MAFRA), pork was the largest source of meat consumption in Korea (49.1%) in 2019. Chicken consumption accounted for 27.1% of Korean meat consumption, and beef accounted for 23.8% in 2019. We examined pork belly prices, average import prices from the U.S. and Australia, and domestic chicken prices. Chilled pork belly was the most popular pork belly in Korea, with a share of approximately 71%. In 2019, 426,000 tons of beef were imported, and 212, 000 tons were produced domestically each year. A total of 682, 000 tons of domestic chicken was consumed annually, which is more than the consumption of imported chicken.

Table 1 presents the descriptive statistics for the meat price variables. We gathered weekly price data for pork, beef, and chicken from the Korea Agricultural Marketing Information Service starting from January 2019 until April 2021 [23]. The average price of pork was 2,079 Korean won (KRW) per 100 g with a standard deviation of 255. The average price of beef was KRW 2, 959 per 100 g, with a standard deviation of 245. The average price of chicken was 2,236 won per kilogram, with a standard deviation of 243. The dataset was matched using the ASF sentiment index. Some ASF news sentiment indexes were removed because meat price data was unavailable during the weekends. Thus, the total sample size was 90.

**Table 1:** Descriptive analysis of variables.

Variable	Obs.	Mean	SD	Min	Max	Units/definition
Pork	90	2,079	255	1,545	2,533	Won per 100 g, chilled pork belly price
Beef	90	,2959	245	2,086	3,316	Won per 100 g, average imported beef price
Chicken	90	5,236	243	4,831	5,889	Domestic chicken price
ASF news sentiment index	90	0.48	0.07	0.32	0.66	

## Results

### ASF news sentiment index

We derived 5,439 words from 24,143 news articles. Next, we filtered them down to 1,015 with 90% cosine similarity (terminating words under 10% relevance) related to the keyword ASF. We then constructed a specified sentiment dictionary for the ASF. We combined 1,015 words selected for the negative side with the Korean Sentiment Lexicon (4863 positive words, 9826 negative words) published by Kunsan National University in South Korea. They proposed a sentiment classification model based on bidirectional long short-term memory (Bi-LSTM) [24]. We secured 4,863 positive and 10,841 negative words for the ASF dictionary.

**Fig 3:**
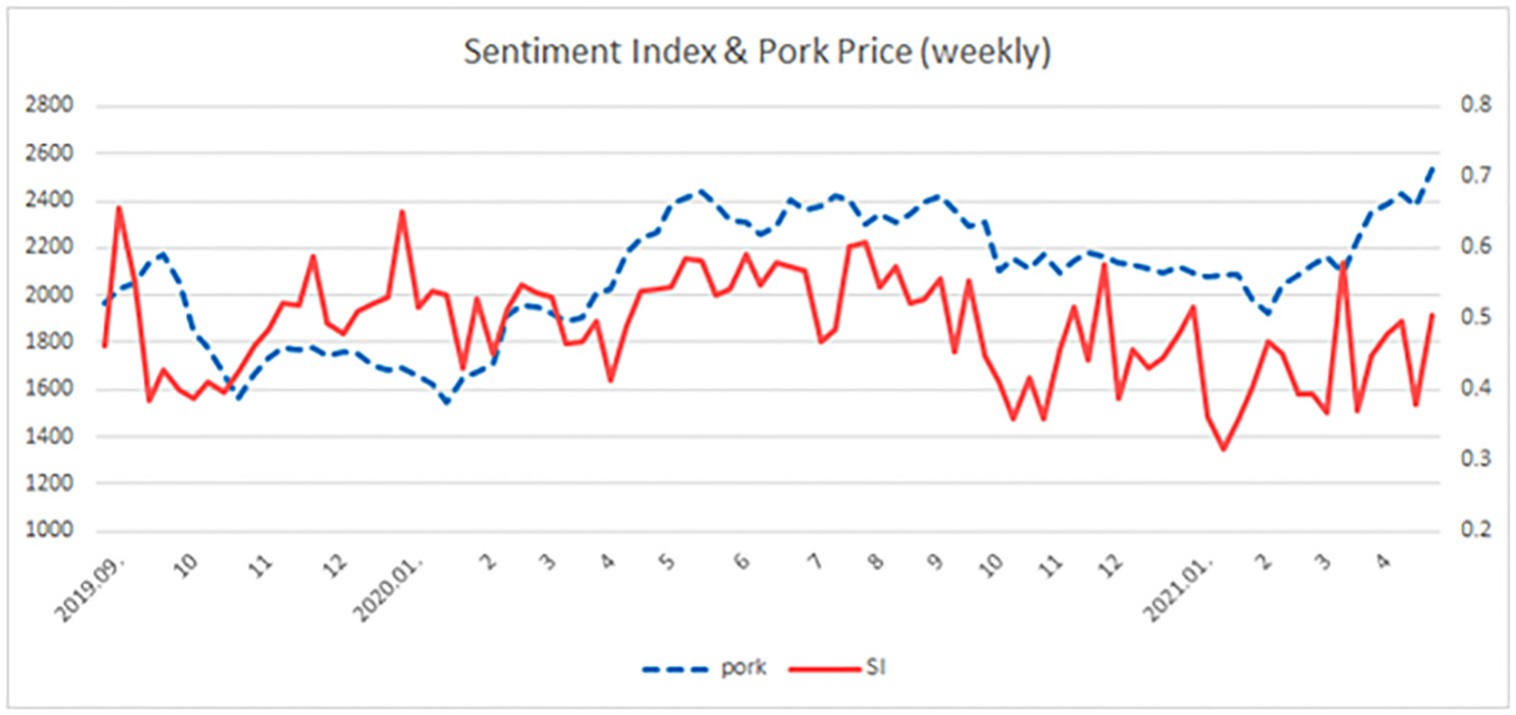
The normalized weekly basis sentiment index. Note: The straight line represents the weekly ASF news sentiment index and the dotted line represents the weekly pork prices. The left y-axis represents pork prices and the right y-axis represents the ASF news sentiment index.

Finally, we summed up the sentence scores by counting the words based on the ASF dictionary and producing a daily and weekly sentiment indexes (see statistics in [S2 Table]). The average ASF news sentiment index was 0.48, which is below 0.5, as shown in Table 1. The fluctuation in the ASF news sentiment index indicates both positive and negative news over time, as shown in Fig 3. When the frequency of ASF news increases, the sentiment index falls below 0.5, indicating that consumers have negative feelings toward ASF news. When the sentiment index is above 0.5, ASF news sentiment is positive. From September to November 2019, ASF was frequently reported, which decreased the ASF news sentiment index to 0.4. In October 2020, an ASF was reported again. At the time, the ASF news sentiment index was less than 0.35. The ASF news sentiment index fluctuates because consumers watched news about possible ASF outbreaks and their prevention.

### Unit root and cointegration test

We tested whether a variable was stationary, as spurious data could be produced [25]. We conducted augmented Dickey–Fuller (ADF) and Kwiatkowski–Phillips–Schmidt–Shin (KPSS) tests to ensure that the data were non-stationary, I(1), or stationary, I(0). The null hypothesis for the ADF test considers the data series to be non-stationary, as shown in Table 2. All data were non-stationary in level and stationary in first difference. For the KPSS, the null hypothesis was that the data were stationary. The KPSS test results showed that the null hypothesis can be rejected at this level but cannot be rejected in the first difference. Therefore, our results imply that all the data have a unit root, namely, I(1), and are non-stationary (Table 2). Therefore, we proceeded to investigate whether the variables have a long-term relationship, using a cointegration test.

**Table 2:** Unit root test results.

	Level		First difference		Stationary
	ADF	KPSS	ADF	KPSS	
Pork	-0.225	0.5623***	-4.0219***	0.0985	I(1)
Beef	0.1678	0.7909***	-6.952***	0.0228	I(1)
Chicken	0.1528	0.2649***	-6.1477***	0.0144	I(1)
SI	-0.0726	0.4502***	-6.7229***	0.0129	I(1)

Notes:

*, **, and *** indicate significance at the 1, 5, and 10% levels, respectively. SI represents the sentiment index of ASF news.

The Johansen test checks whether several non-stationary variables are cointegrated. We used the multivariate cointegration test procedure developed by Johansen, instead of the bivariate cointegration test, as we considered more than two variables. As shown in Table 3, the null hypothesis that there is no cointegration is rejected at the 5% critical level, both in the trace and the eigenvalue statistics. We find at most one cointegration among the variables, and the results indicated that some variables move together in the long run. Hence, we used the vector error correction model (VECM) to yield more efficient estimates [26]. The optimal lag was chosen based on the AIC criterion, which indicated that the optimal lag order was two.

**Table 3:** Johansen cointegration test results.

Null hypothesis	Trace statistic	5% critical value	Maximum eigenvalue statistic	5% critical value
r = 0	140.49	53.12	89.34	28.14
r = <1*	51.15	34.91	31.84	22
r = <2	19.31	19.96	16.10	15.67
r = <3	3.20	9.24	3.20	9.24

Notes:

* indicates rejection of the hypothesis at the 5% level.

### Impulse response and variance decomposition

**Fig 4:**
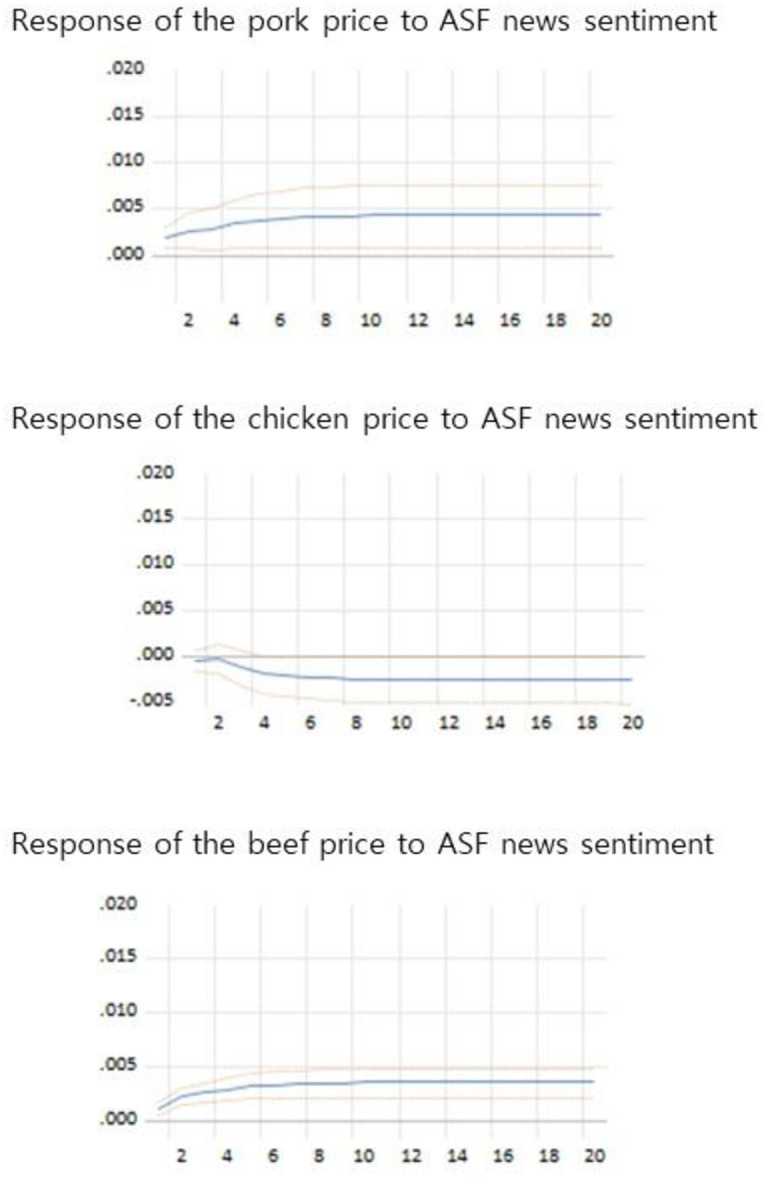
Impulse response graph. Note: The first, second, and third graphs show the responses of pork, chicken, and beef prices to the sentiment index, respectively.

The results of the impulse response test, for understanding the effect of ASF news shock on pork, beef, and chicken prices are shown in Fig 4. Each figure shows the effect of a one-standard-deviation structural change in the ASF news shock, on present and future meat prices. Therefore, the ASF news shock in Fig 4 positively impacts meat prices. The solid line represents the impulse response and the dotted lines represent the 90% confidence bands. All the results of impulse response were statistically significant at the 90% confidence level. The response time horizon was set as 10 days.

A positive ASF news shock increased pork prices. A shock of one standard deviation to ASF news sentiment caused a significant increase in pork prices for several days. From Eq (4), we found that price elasticity with respect to ASF news for pork was positive. This result implied that the demand elasticity with respect to ASF news, was greater than that of the supply elasticity. In other words, the change in demand in response to positive ASF news, dominated the increase in supply.

However, a negative ASF news shock lowered pork prices because the shift in demand was greater than the shift in supply. It meant that, the buyers were apprehensive about consuming pork during the ASF outbreak. Hence, if consumers saw positive ASF news, such as a reduction in ASF outbreaks, or that related to fewer or no health risks associated with the ASF outbreak, pork consumption would increase. Our findings align with prior research, including Ishida et al. [2], which indicates that outbreaks such as BSE and bird flu have led to reduced consumption of beef and chicken due to consumer health concerns.

We obtained different responses regarding prices in the beef and pork markets. Positive ASF news shocks lowered the chicken prices. We know that the response of the pork price to the ASF news shock is positive; therefore, $\varepsilon_{p_{p},I}^{i}$ in Eq (6) is positive. We presumed that a positive ASF news shock would decrease chicken consumption and production. Hence, in Eq (6), the demand elasticities with respect to ASF news for chicken would be larger than the supply elasticities, in absolute values. This would mean that chicken production would decrease, because producers expect pork consumption to be substituted with chicken. However, consumers’ responses to positive ASF news were relatively large compared with producers’ responses. Positive ASF news can reduce beef production, as producers can expect an increase in pork consumption; however, there was little impact on beef consumption. Thus, a positive ASF news shock increased beef prices.

The impact of ASF news on meat prices can last up to 10 days. The increase in the pork prices lasted 10 days and chicken prices remained the same after the positive ASF news shock. The price of beef increased slightly and then remained constant a few days later. Therefore, ASF news sentiment directly affected pork prices for a longer period of time than other meat prices. Moreover, ASF news sentiment indirectly affects the prices of beef and chicken; while the beef prices increased, the chicken prices decreased.

The variance decompositions (VD) of meat prices reported in Table 4 examined the influence of ASF news sentiment on meat prices. The VD results showed that ASF news sentiment largely influenced pork prices, compared with beef and chicken prices. The impact of ASF news shock accounted for 2.25% of the variation in the fluctuation in the pork prices in the five periods; and increased by 1.33% points across the 10 periods. The ASF news shocks have a relatively small impact on chicken prices. The ASF news generated a 0.24% variation in the fluctuation in the chicken prices in the five periods, and 0.66% in the 10 periods. Furthermore, the ASF news influenced beef prices. Compared to chicken prices, the price of beef was largely influenced by ASF news sentiment, which meant that beef was strongly substituted with pork on the supply side.

**Table 4:** Variance decompositions of meat price variables to the ASF news.

Period	Chicken	Beef	Pork	Sentiment index
Variance decomposition of pork price:				
1	0.53	0.37	98.09	1.00
5	0.51	0.53	96.68	2.25
10	0.61	0.66	95.14	3.58
Variance decomposition of chicken price:				
1	100.0	0.00	0.00	0.00
5	98.83	0.73	0.18	0.24
10	98.28	0.75	0.28	0.66
Variance decomposition of beef price:				
1	0.34	99.65	0.00	0.00
5	0.10	97.75	0.51	1.62
10	0.09	95.54	0.58	3.76

## Conclusions

ASF news sentiment played a key role in changing consumer behavior toward meat products. Consumers watched ASF news in positive or negative moods and were likely to change their meat consumption behavior. Therefore, we examined the impact of ASF news sentiment on meat prices in Korea. First, we used NNLM to generate the ASF sentiment index. We derived 5,439 words from 24,143 news titles and generated an ASF lexicon consisting of 4,870 positive and 10,264 negative words. The IRF in the VECM system, comprised the ASF news sentiment index and pork, beef, and chicken prices, and was used to examine the response of meat prices to the ASF news sentiment shock.

ASF news sentiment ranged from 0 to 1. We generated weekly ASF news sentiment from September 2019 to May 2021. An ASF news sentiment index greater than 0.5 was interpreted as the positive mode, and less than 0.5, as the negative mode. We found that the average ASF news sentiment value was below 0.5, indicating that consumers had negative feelings toward ASF news. Additionally, when the ASF outbreak occurred, the ASF news sentiment decreased to 0.35. The occurrence of ASF over time created fluctuations in the sentiment index, and this could in turn affect meat consumption behavior in Korea. This change in ASF news sentiments could affect meat consumption behavior in Korea.

The impulse response results suggested that ASF news sentiment significantly affected meat prices in South Korea. When the substitute effects were considered, positive ASF news sentiment raised pork and beef prices, whereas chicken prices decreased. This response implied that the demand change created by positive ASF news dominated the supply changes. Moreover, the chicken price response to positive ASF news was the opposite to the beef price response, indicating that chicken consumption and beef production were strongly substituted with pork. In addition, VD supports a strong relationship between pork and chicken consumption. The results indicated that ASF news largely influenced pork and chicken prices compared with other prices.

This study has some limitations. First, news about viruses and diseases incites criticism and people are easily exposed to negative sentiments. We argue that negative sentiments play a crucial role in determining the social impact. Second, the Korean BERT dataset conformed to the analysis of SNS reviews (Korean website Naver movie review dataset), indicating a poor level of supervised learning results. Before creating the BERT dataset for news titles, the research must focus on word-level sentiment analysis. Hence, there is room for improvement, including the application of other machine-learning methods. Finally, we did not capture the response of meat consumption to ASF news sentiments because of the lack of weekly time-series data on meat quantities. Nevertheless, our study provides policy implications that the ASF outbreak affects the pork, beef, and chicken markets. This evidence suggests that policymakers should consider the meat market when producers and consumers face an ASF outbreak. Moreover, our study is more likely to introduce agricultural economists to estimate the impact of ASF related news on meat markets. Our study demonstrates the value of hybrid research approaches in enhancing the understanding of complex economic systems and highlights the potential for further research in this field. Our methods and results may inspire discussion among applied economists studying this market, and could encourage the application of big data analysis to the agricultural economy.

## Supporting information

S1 Table ASF news frequency in daily. (DOCX)

S2 Table Summary of statistics about sentiment score. (DOCX)

S1 File Measuring similarity with cosine of the angle. (DOCX)

S2 File The test of embedding result on ASF. (DOCX)

S3 File Flow chart of ‘KNU sentiment lexicon’ construct algorithms. (DOCX)

S4 File General structure of bidirectional recurrent neural network. (DOCX)

S1 Data Original news data. (XLSX)

S2 Data Sc score and frequency. (XLSX)

S3 Data Sc index. (XLSX)
