# Supplementary material for: The impact of African swine fever news sentiment on the Korean meat market
Source: PLoS One. 2023 Jun 30;18(6):e0286520. doi: 10.1371/journal.pone.0286520 (PMC10313005; doi:10.1371/journal.pone.0286520)
Supplement: S1 Table — (DOCX) [file pone.0286520.s001.docx]

S1 Table. ASF News Frequency in Daily

| ASF News Frequency in Daily | |
| --- | --- |
|  |  |
| Average | 29.66 |
| Standard Error | 2.35 |
| Median | 13 |
| Mode | 2 |
| Standard deviation | 67.07 |
| Sample variance | 4498.70 |
| Kurtosis | 53.42 |
| Skewness | 6.48 |
| Range | 823 |
| Minimum | 1 |
| Maximum | 824 |
| Sum | 24143 |
| Count | 814 |
|  |  |

*The table produces daily-level frequency for 24,143 articles about African Swine Fever over 814 days.
