# Supplementary material for: The impact of African swine fever news sentiment on the Korean meat market
Source: PLoS One. 2023 Jun 30;18(6):e0286520. doi: 10.1371/journal.pone.0286520 (PMC10313005; doi:10.1371/journal.pone.0286520)
Supplement: S2 Table — (DOCX) [file pone.0286520.s002.docx]

S2 Table. Summary of statistics about sentiment score

| Sentiment score | |
| --- | --- |
|  |  |
| Average | -1.03 |
| Standard Error | 0.01 |
| Median | -1 |
| Mode | 0 |
| Standard deviation | 1.20 |
| Sample variance | 1.43 |
| Kurtosis | 0.38 |
| Skewness | -0.69 |
| Range | 12 |
| Minimum | -7 |
| Maximum | 5 |
| Sum | -24808 |
| Count | 24143 |
