# Supplementary material for: The impact of African swine fever news sentiment on the Korean meat market
Source: PLoS One. 2023 Jun 30;18(6):e0286520. doi: 10.1371/journal.pone.0286520 (PMC10313005; doi:10.1371/journal.pone.0286520)
Supplement: S1 File — (DOCX) [file pone.0286520.s003.docx]

S1 File. Measuring Similarity With Cosine of the Angle


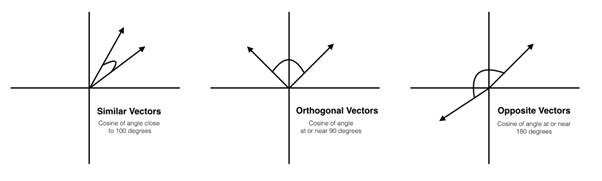


Tellez et al. (2017) present cosine similarity. The similarity has a value over -1 to 1, and this study derives that “Pig” has the value of 0.94 with “Africa” (0.96 with “Fever”). Africa is not a negative word in general, on the other hand in ASF news, “Africa” could be a cynical word. It implies that cosine similarity draws not the only similarity of meaning but the relevance of meaning. Cosine similarity helps to analyze the contextual meaning of news sentiment.
