# Supplementary material for: The impact of African swine fever news sentiment on the Korean meat market
Source: PLoS One. 2023 Jun 30;18(6):e0286520. doi: 10.1371/journal.pone.0286520 (PMC10313005; doi:10.1371/journal.pone.0286520)
Supplement: S2 File — (DOCX) [file pone.0286520.s004.docx]

S2 File. The Test of Embedding Result on ASF

We tested the quality of embedding results on ASF. Each word allocated the value in 100 dimensions. It means that an embedded word has a unique value and then arithmetically calculates the relationship between words. We introduce an example as follows,


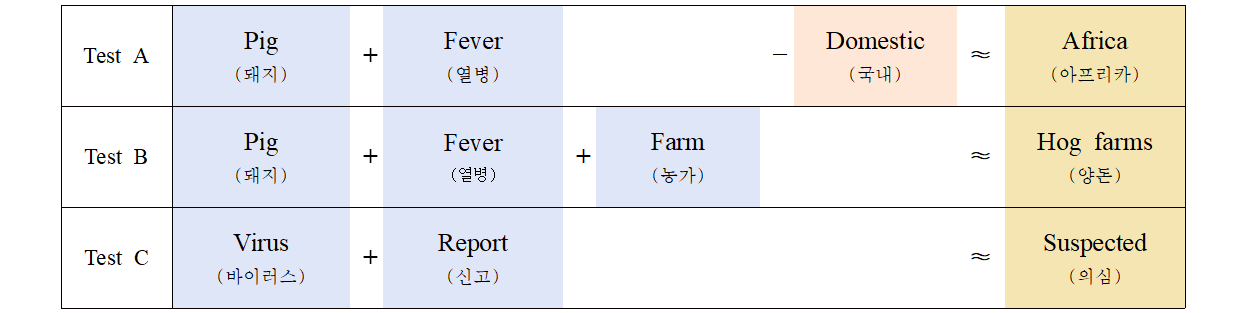


* ( ) = Korean

The table gives an intuitive explanation of the result. In Test A, when the “Pig” and “Fever” are added and the “Domestic” is excluded, the word “Africa” is derived. It is reasonable since the ASF outbreak was in place in Korea and damaged hog farms first in 2019. Moreover, in Test C, we found that the embedding results provide a contextual outcome, which means “Virus” plus “Report” equal to “Suspected”.


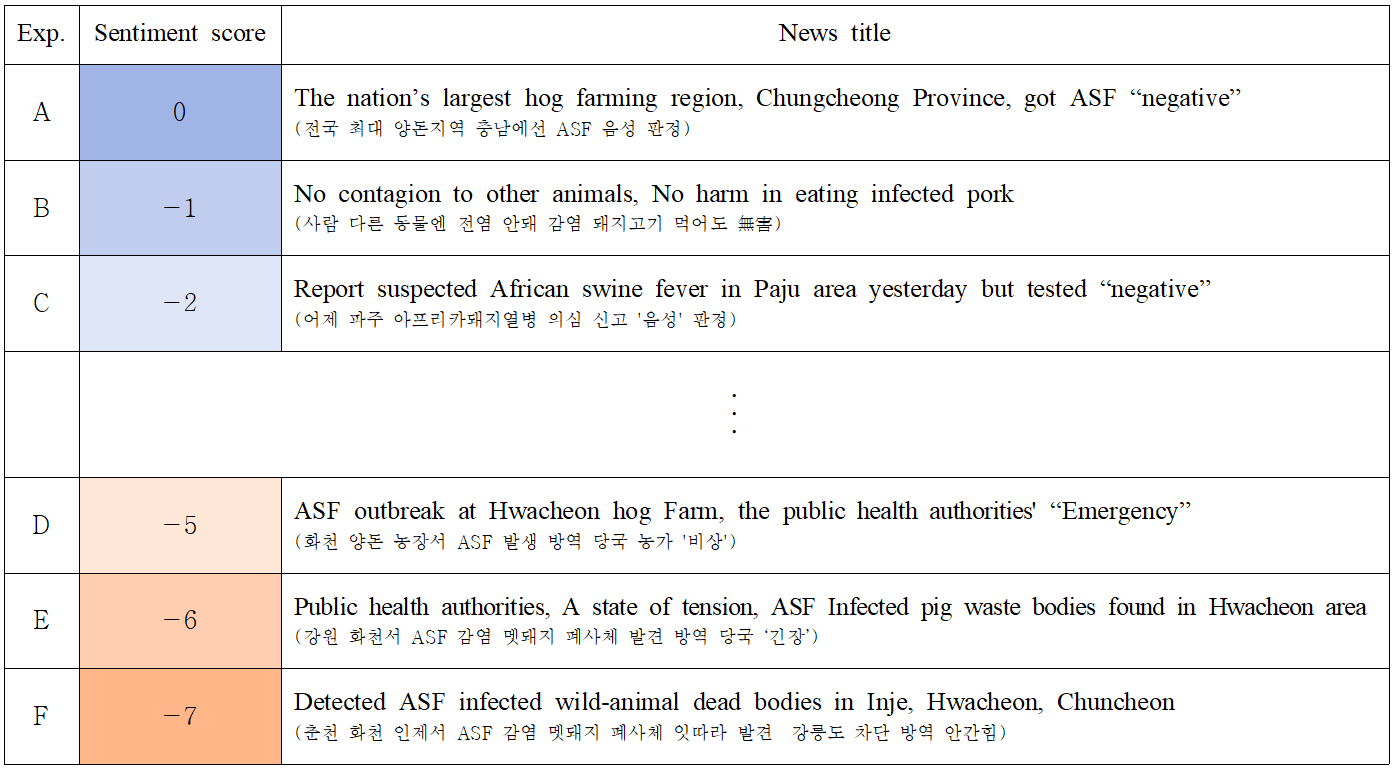


* ( ) = Korean

We provide some examples above A to F. There is sentence level driven by embedded words. Even though the upper case (A, B, C) includes ASF, African, fever; it draws a lower sentiment score than the bottom case (D, E, F). In the table, we identify the intent of context in news titles and reasonably derive negativity.
