# Supplementary material for: The impact of African swine fever news sentiment on the Korean meat market
Source: PLoS One. 2023 Jun 30;18(6):e0286520. doi: 10.1371/journal.pone.0286520 (PMC10313005; doi:10.1371/journal.pone.0286520)
Supplement: S3 File — (DOCX) [file pone.0286520.s005.docx]

S3 File. Flow chart of ‘KNU Sentiment Lexicon’ construct algorithms


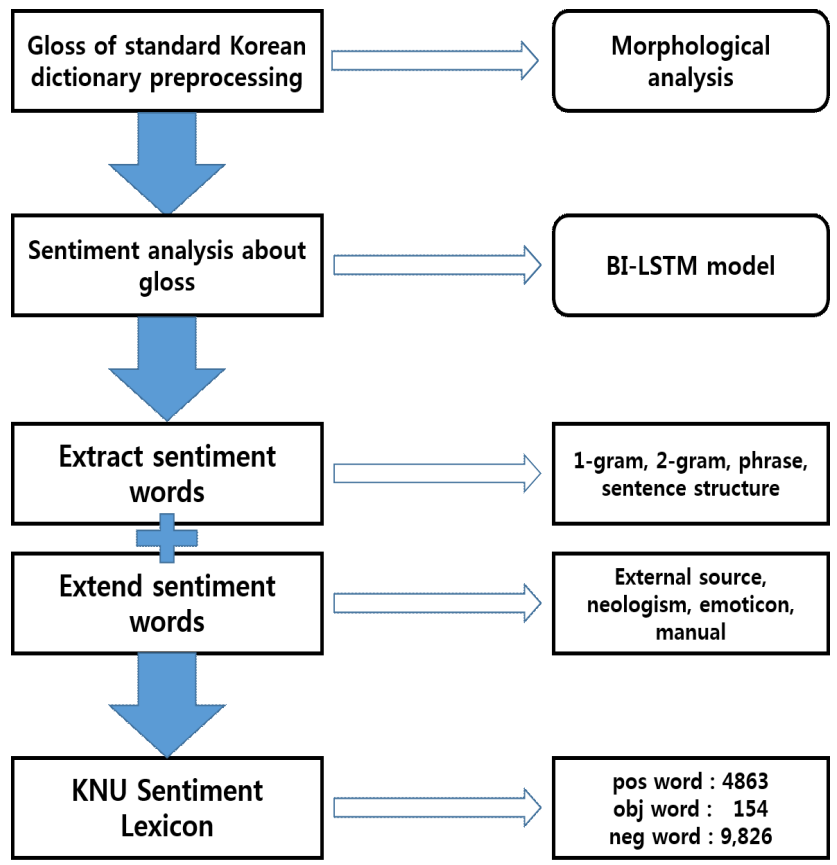


Note: Adapted from Park et al. (2018).

KNU sentiment lexicon is attributed to the word-level NLP method of creating a Korean sentiment word dictionary. Above Fig 5. shows the flow of construction of the KNU sentiment lexicon. It is derived from the ‘Standard Korean Language Dictionary (SKLD)’ published by National Institute of the Korean Language(NIKL). All of the words in SKLD were scraped and tokenized to match with gloss. They assume that if the gloss shows positive sentiment, sentence contains at least one positive word. Three voters made dataset for gloss by hand. If all voters agree to the same sentiment for a gloss(sentence), a gloss is added to dataset, or not, it is excluded from dataset.
