# Supplementary material for: The impact of African swine fever news sentiment on the Korean meat market
Source: PLoS One. 2023 Jun 30;18(6):e0286520. doi: 10.1371/journal.pone.0286520 (PMC10313005; doi:10.1371/journal.pone.0286520)
Supplement: S4 File — (DOCX) [file pone.0286520.s006.docx]

S4 File. General structure of bidirectional recurrent neural network


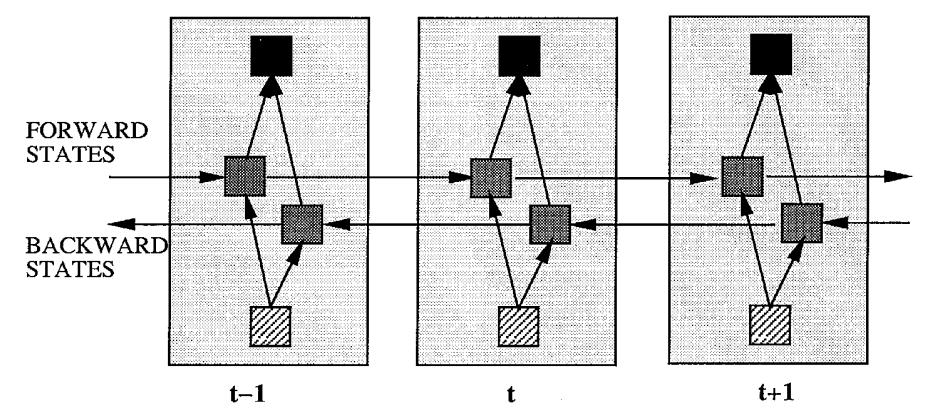


Note: Adapted from Shcuster and Plaiwal (1997)

The dataset has been trained by Bidirectional-LSTM(Bi-LSTM). LSTM is one of recurrent neural networks to analyze sequential data. Shcuster and Paliwal (1997) proposed Bi-RNNs that powerful tools for considering forward and backward states at once (Fig 6.) [18]. Hidden state in Bi-LSTM represents both left and right context. Based on the trained result, they extracted sentiment words (1-gram, 2-gram, phrase, sentence structure) from SKLD and created ‘KNU sentiment dictionary'. Moreover, it has extended with external sources, emoticons, and so on. Finally, the dictionary contains 4,863 positive words and 9,826 negative words.
